# Heme Oxygenase-1 Expression Affects Murine Abdominal Aortic Aneurysm Progression

**DOI:** 10.1371/journal.pone.0149288

**Published:** 2016-02-19

**Authors:** Junya Azuma, Ronald J. Wong, Takeshi Morisawa, Mark Hsu, Lars Maegdefessel, Hui Zhao, Flora Kalish, Yosuke Kayama, Matthew B. Wallenstein, Alicia C. Deng, Joshua M. Spin, David K. Stevenson, Ronald L. Dalman, Philip S. Tsao

**Affiliations:** 1 Division of Cardiovascular Medicine, Stanford University School of Medicine, Stanford, CA, United States of America; 2 Department of Pediatrics, Division of Neonatal and Developmental Medicine, Stanford University School of Medicine, Stanford, CA, United States of America; 3 Division of Vascular Surgery, Stanford University School of Medicine, Stanford, CA, United States of America; 4 Veterans Affairs Palo Alto Health Care System, Palo Alto, CA, United States of America; Max-Delbrück Center for Molecular Medicine (MDC), GERMANY

## Abstract

Heme oxygenase-1 (HO-1), the rate-limiting enzyme in heme degradation, is a cytoprotective enzyme upregulated in the vasculature by increased flow and inflammatory stimuli. Human genetic data suggest that a diminished HO-1 expression may predispose one to abdominal aortic aneurysm (AAA) development. In addition, heme is known to strongly induce HO-1 expression. Utilizing the porcine pancreatic elastase (PPE) model of AAA induction in HO-1 heterozygous (HO-1^+/-^, HO-1 Het) mice, we found that a deficiency in HO-1 leads to augmented AAA development. Peritoneal macrophages from HO-1^+/-^ mice showed increased gene expression of pro-inflammatory cytokines, including MCP-1, TNF-alpha, IL-1-beta, and IL-6, but decreased expression of anti-inflammatory cytokines IL-10 and TGF-beta. Furthermore, treatment with heme returned AAA progression in HO-1 Het mice to a wild-type profile. Using a second murine AAA model (Ang II-ApoE^-/-^), we showed that low doses of the HMG-CoA reductase inhibitor rosuvastatin can induce HO-1 expression in aortic tissue and suppress AAA progression in the absence of lipid lowering. Our results support those studies that suggest that pleiotropic statin effects might be beneficial in AAA, possibly through the upregulation of HO-1. Specific targeted therapies designed to induce HO-1 could become an adjunctive therapeutic strategy for the prevention of AAA disease.

## Introduction

Abdominal aortic aneurysm (AAA) disease is a potentially lethal condition that is characterized by the destructive remodeling of the infrarenal (IR) aorta [1]. It is a complex disease process involving the infiltration of inflammatory cells, the production of reactive oxygen species (ROS), upregulation and activation of degradative proteases, inactivation of nascent protease inhibitors, stimulation of apoptosis, degradation of elastin, transmural inflammation, and resistive hemodynamic conditions [2]. Currently, there is no effective medical or pharmaceutical intervention available for small AAAs that might delay or prevent the need for invasive aneurysm repair.

One mechanism of particular interest is the flow-related upregulation of an anti-inflammatory enzyme, heme oxygenase-1 (HO-1). Polymorphisms in the HO-1 promoter region have been linked to AAA disease risk in humans [3]. Three isoforms of HO have been described. Two (HO-2 and HO-3) are constitutively expressed, while HO-1 is inducible by inflammatory stimuli. HO-1 degrades heme and produces equimolar concentrations of the bioactive products: carbon monoxide (CO), which is a vasodilator, ferrous iron, and biliverdin, which is rapidly converted to bilirubin by biliverdin reductase. Biliverdin and bilirubin are potent antioxidants [4,5] and can protect the vascular endothelium and intima-media, and attenuate lesion formation following injury. Other potential protective effects of HO-1 include a reduction of vascular smooth muscle cell (VSMC) proliferation, attenuation of vasoconstriction [6], scavenging of free radicals, inhibition of platelet aggregation [7], and up-regulation of transferrin expression with subsequent sequestration of oxidative free iron [8]. Although high levels of HO activity (>15-fold) produce toxic, reactive iron species, moderate increases (<5-fold) appear to be cytoprotective in animal models [9]. It has been shown that heme administration can strongly upregulate HO-1 activity [10,11]. An increase in HO-1 gene expression has been observed under high-flow conditions and associated with experimental AAA attenuation [12].

3-hydroxy-3-methyl-glutaryl-CoA reductase (HMG-CoA reductase) inhibitors (or statins) are lipid-lowering agents that are widely used clinically [13]. Reduction of plasma lipids through statins occurs by competitive inhibition of HMG-CoA reductase, an enzyme crucial for cholesterol synthesis. Mechanisms beyond the reduction of plasma cholesterol levels contribute significantly to the anti-atherogenic and tissue protective properties of statins. These pleiotropic, cholesterol-independent benefits include anti-proliferative [14], immunomodulatory [14], vascular cytoprotection [15], angiogenic [16], plaque stabilizing [17,18], and antioxidative effects [14,19], and anti-inflammatory properties [19,20]. While some trials have seen no improvement in AAA progression in patients undergoing statin therapy [21], observational studies and a meta-analysis suggest that statin therapy is associated with decreased expansion rates in patients with small AAAs [22]. We previously demonstrated that statins induce HO-1 expression and reduce oxidative stress in vascular cells in vivo [23,24]. We hypothesize that these properties of HO-1 may be one of the therapeutic effects of statins in ameliorating the development of AAA disease.

## Materials and Methods

### Animals

Eight-week-old apolipoprotein E-deficient (ApoE^-/-^) male mice (n = 18) with a C57BL/6J background were purchased from Jackson Laboratories (Bar Harbor, ME). In our second set of studies, 8-wk-old wild-type (WT, HO-1^+/+^, n = 14) and HO-1 heterozygous (HO-1 Het, HO-1^+/-^, n = 13) FVB mice were used and purchased from Jackson Laboratories. For studies investigating the effect of AAA development on HO-1 promoter activity, we used adult HO-1-*luc* mice (8-wk-old), whose transgene contains the full-length HO-1 promoter fused to the reported gene luciferase. All mice were provided with water and food ad libitum. All studies were approved by the Stanford University Institutional Animal Care and Use Committee.

### Angiotensin II-ApoE^-/-^ AAA model

The subcutaneous osmotic angiotensin II (Ang II) infusion model was used to create suprarenal (SR) murine AAAs in the ApoE^-/-^ mice. Under inhaled anesthesia with 2% isoflurane, osmotic mini-pumps (Alzet Model 2004, Durect Corp., Cupertino, CA), prepared in sterile manner with Ang II (Sigma-Aldrich, St. Louis, MO) in saline, were inserted beneath the dorsal skin of each mouse. Pumps were set to deliver Ang II at constant rate of 1000 ng/kg/min [25,26]. All mice were then monitored daily for up to 28 days.

### Porcine pancreatic elastase AAA model–HO-1^+/-^ and C57Bl6 mice

The porcine pancreatic elastase (PPE) infusion model was used to induce IR AAAs as previously described [27] in WT and HO-1 Het FVB mice. In brief, after placing temporary ligatures around the proximal and distal aortae, an aortotomy was created at the bifurcation, and a catheter was inserted into the aorta and saline or saline containing type I PPE (1.5 U/mL, Sigma-Aldrich) were infused for 5 min at 100 mmHg. After removing the infusion catheter, the aortotomy was repaired without constriction of the lumen. The health and condition of all mice were monitored daily. At 14 or 28 days, the induced AAA (area between the left renal artery and the bifurcation) was harvested. Samples were snap frozen in liquid nitrogen and then stored at –80°C pending further processing. The survival rate through day 28 was > 80% for all experimental groups. In those mice that did not survive, no clinical signs of ill health were observed prior to death.

### Statin treatment and experimental design–Ang II model

Rosuvastatin (AstraZeneca, Wilmington, DE) was dissolved in 30-μL polysorbate 80. Normal saline was added to yield 100 mg/kg stock solutions. The saline vehicle was prepared similarly without the addition of rosuvastatin. The dose of rosuvastatin was chosen to elicit pleiotropic effects in the absence of lipid lowering as previously reported [23,24]. ApoE^-/-^ mice undergoing Ang II infusion were randomized and treated with daily intraperitoneal (IP) injections of saline vehicle (n = 8) or rosuvastatin (n = 10) for 35 days, beginning 21 days prior to pump implantation. After this period of time, all animals were sacrificed and post-mortem measurements of suprarenal (SR) and IR aortic diameters were performed. AAA severity was classified as follows: Type 1, SR/IR ratio < 2; Type 2, SR/IR ratio > 2; Type 3, presence of aortic dissection. Measurements of serum total cholesterol, triglycerides, high-density lipoprotein (HDL), and low-density lipoprotein (LDL) were performed using enzymatic assay kits.

### Heme treatment–PPE model

HO-1 Het mice included in the PPE infusion AAA model were randomized to receive an IP injection of vehicle (n = 6) or heme (60 μmol/kg BW, n = 7), which is a potent inducer of HO-1 [10,11], 1 day prior to and 14 days after PPE infusion.

### HO activity–AAA models

Measurements of HO activity in both Ang II- and PPE-treated mice were performed as previously described [24,28]. Briefly, aortae were harvested following sacrifice, diluted in phosphate buffer (1+9 v:v), diced with scissors, and sonicated at 50% power using a Microson Ultrasonic Cell Disruptor (Misonix, Farmingdale, NY). Sonicates [20 μL, representing 2 mg fresh weight (FW)] were incubated in CO-free septum-sealed vials containing 20 μL of 150-μM methemalbumin (MHA) and 20 μL of 1.5-mM NADPH (blank reaction contained phosphate in place of NADPH) for 30 min. Reactions were then terminated with the addition of 5 μL of 30% sulfosalicylic acid (SSA, Sigma-Aldrich). The amount of CO in the vial headspace was then quantified by gas chromatography (GC). HO activity was calculated as pmol CO/h/mg FW, expressed as fold change from saline-treated controls, and compared between all groups.

### Monitoring of AAA progression

Under inhaled anesthesia (2% isoflurane), maximum diameters in the transverse plane of the aortae of all mice employed in the AAA model experiments were imaged using a Vevo 770 High-Resolution In Vivo Micro-Imaging System (VisualSonics, Toronto, CN) fitted with a 40-MHz transducer while placed in the supine position. The IR and SR aortae of rosuvastatin-treated and control mice were measured at 0 and 14 days. The SR aortae were imaged prior to pump implantation, AAA induction at day 0 and then at 7, 14, 21 and 28 days post-operatively, Briefly, mice were first anesthetized with 5% isoflurane in 1 L O_2_/min and then maintained at 3% isoflurane in 1 L O_2_/min for the duration of the scan. Mice were kept warm on a heated platform (37±0.5°C) connected to a temperature/physiology monitor (Indus Instrument, Houston TX). Fur from the abdominal region was removed using a commercial hair remover (Nair) and Ultrasound Transmission Gel (Parker Laboratories Inc., Fairfield, NJ) applied to this area. The Power-Doppler mode was used to monitor blood flow velocities and pulse rates. During the entire procedure, heart rates and rhythms, respirations, and body temperatures were recorded. Aortic diameters were measured using transabdominal ultrasonography. Baseline measurements were obtained 1 day prior to PPE infusion, and then every 7 days through study completion at 28 days post-PPE infusion.

### Immunohistochemical studies

Immunohistochemistry was performed to identify the cells expressing Mac1 and HO-1 proteins. 6-μM thick sections were cut from paraffin-embedded aortae. After deparaffinization, sections were treated for endogenous peroxidases with 0.03% H_2_O_2_ and blocked in 1% BSA and 5% normal goat serum, then incubated with a primary antibody against Mac1 (1:200, Abcam, Cambridge, MA) or HO-1 followed by the anti-rabbit immunoglobulin G (IgG) secondary antibody (1:200, BA-1000; Vector Labs, Burlingame, CA). Antibodies were then detected using the Vector ABC kit (Elite Vectastain ABC kit; Vector Labs) and colorized with 0.05% diaminobenzidine (DAB; Vector Labs). Negative controls were run in parallel using adjacent sections incubated with IgG instead of the primary antibody.

### Thioglycollate-elicited macrophages and HO activity *in vitro*

Five days after IP injection of 3-mL 3% thioglycollate, primary peritoneal macrophages were harvested from age-matched WT and HO-1 Het mice via IP lavage with phosphate-buffered saline. Cells were plated in 10% fetal bovine serum with Dulbecco’s modified eagle medium with 1% penicillin-streptomycin in 6-well plates. Cells were harvested for RNA isolation 6h after plating.

### RNA quantification

Total RNA was isolated using a TRIzol-based (Invitrogen, Carlsbad, CA) RNA isolation protocol and was quantified by Nanodrop (Agilent Technologies, Santa Clara, CA). Samples required 260/280 ratios >1.8. The iScript cDNA synthesis kit (Bio-Rad) was used to synthesize first-strand cDNA according to the manufacturer’s protocol. TaqMan qRT-PCR assays were performed using mouse-specific primers (Applied Biosystems Inc., Foster City, CA). All probes were normalized to 18S as a multiplexed internal control. Amplification took place on either a PRISM 7900HT or a QuantStudio12K Flex (Applied Biosystems). All fold changes were calculated by the method of ΔΔCt, and are expressed as mean±SD compared to controls. All experiments included 5 to 12 samples per group and time point.

### Statistical analyses

Data were presented as mean±SD and were analyzed using unpaired t-tests and non-parametric Mann-Whitney tests for the in vivo studies. Statistical significance was set at p<0.05.

## Results

### Diminished HO-1 activity increases AAA and increases macrophage infiltration

**Fig 1:**
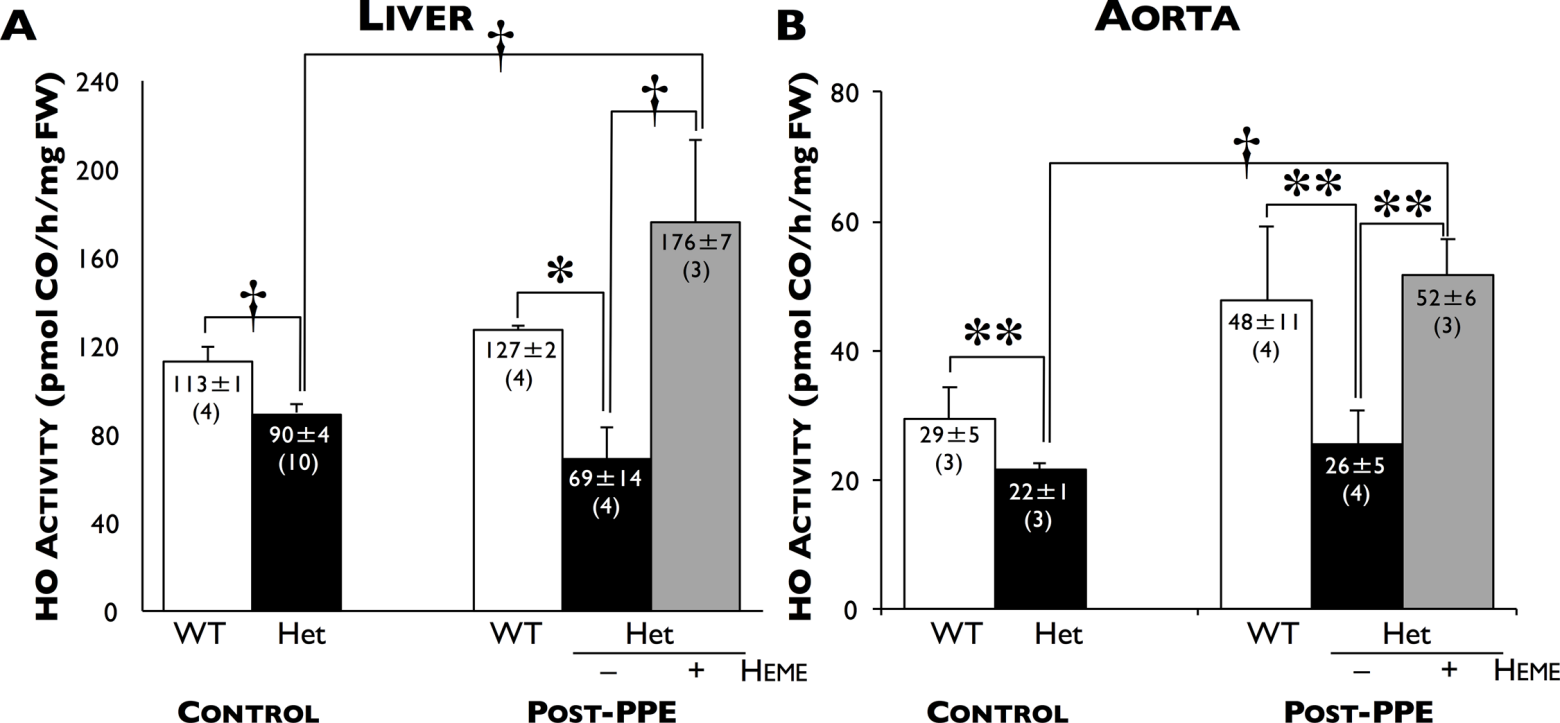
HO activity. Total HO enzyme activity (pmol CO/h/mg FW) in the (**A**) livers and (**B**) aortae of WT (white bars), HO-1 Het (black bars), and heme-treated HO-1 Het (grey bars) mice 28 days after PPE infusion (Post-PPE) and age-matched WT and HO-1 Het controls. *p≤0.006, †p<0.025, **p≤0.05. Number of animals in each group are shown in parentheses.

As expected, total basal HO activity in the livers (90±4 pmol CO/h/mg FW, n = 10) and aortae (22±1 pmol CO/h/mg FW, n = 3) of HO-1 Het mice were significantly lower compared to WT mice (113±1, n = 4, p≤0.006 and 29±5 pmol CO/h/mg FW, n = 3, p≤0.05, respectively, Fig 1A and 1B, left panels). After PPE infusion, there were significant increases in HO activity only in the livers (176±7 pmol CO/h/mg FW, n = 3) and aortae (52±6 pmol CO/h/mg FW, n = 4) of heme-treated HO-1 Het mice compared to HO-1 Het controls (p≤0.025).

**Fig 2:**
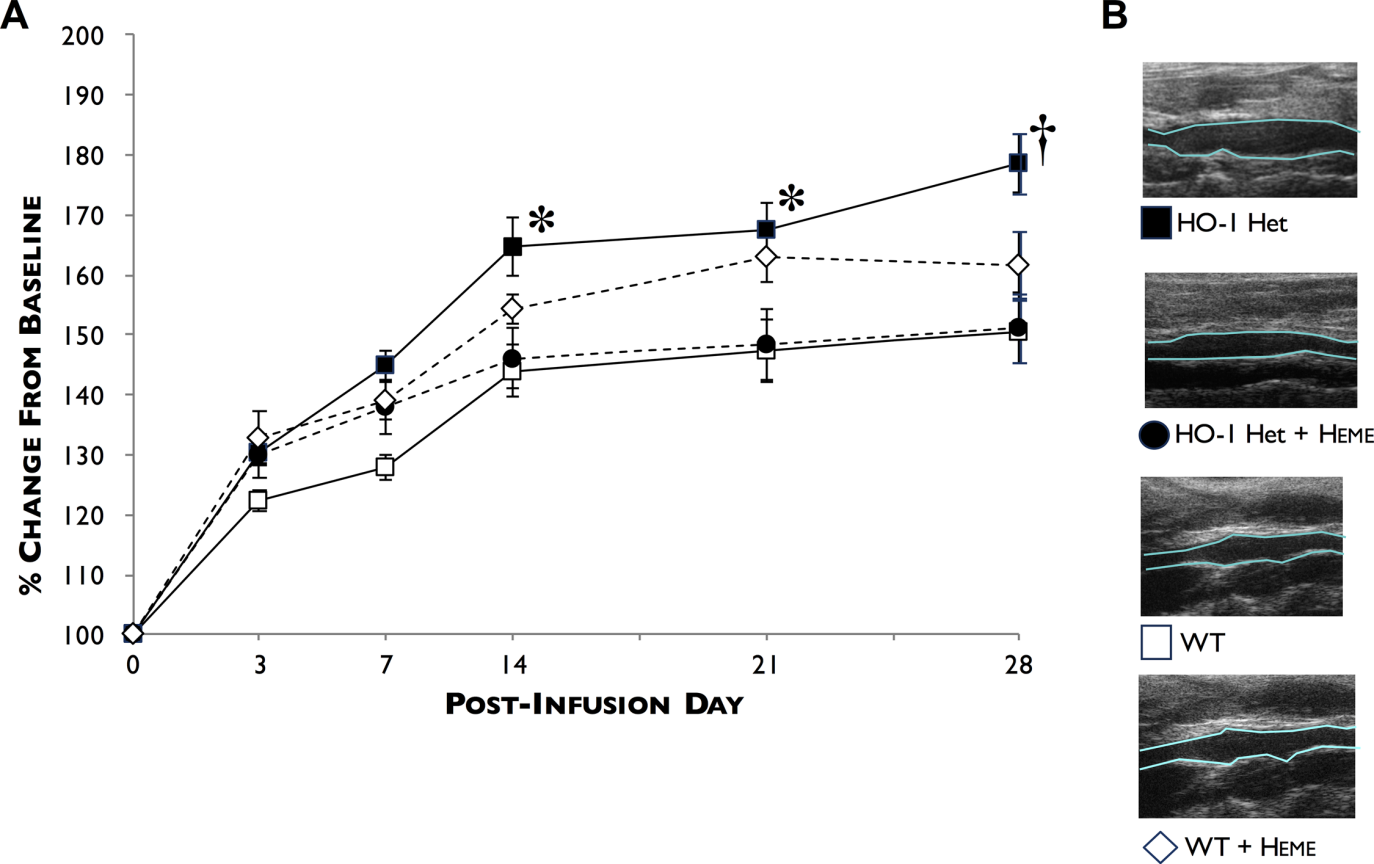
Profiles of AAA development following PPE infusion. (**A**) AAA diameters were measured at baseline and at 3, 7, 14, 21, and 28 days post-PPE infusion by ultrasound in WT (white squares), HO-1 Het (black squares), heme-treated WT (white diamonds), and heme-treated HO-1 Het (black circles) mice. (**B**) Representative longitudinal ultrasound views of AAAs 28-days post-PPE infusion from HO-1 Het, heme-treated HO-1 Het, WT, and heme-treated WT mice. *p<0.015, †p = 0.0025 compared to WT and heme-treated HO-1 Het mice. n = 7 to 11 for each group.

Fig 2 shows the rate of abdominal aortic diameter enlargement after PPE infusion at days 3, 7, 14, 21, and 28 post-PPE infusion. We found that the size of aortic diameters significantly increased in HO-1 Het mice compared to WT mice beginning day 7, and progressively significantly increasing from days 14, 21, and 28 (45.0±7.6%, 64.8±15.3% (p = 0.010), 67.7±13.4% (p<0.015), and 78.6±15.8% (p<0.003) for HO-1 Het mice *versus* 27.9±6.5%, 44.1±12.8%, 47.5±15.8%, and 50.5±16.0% for WT mice.

**Fig 3:**
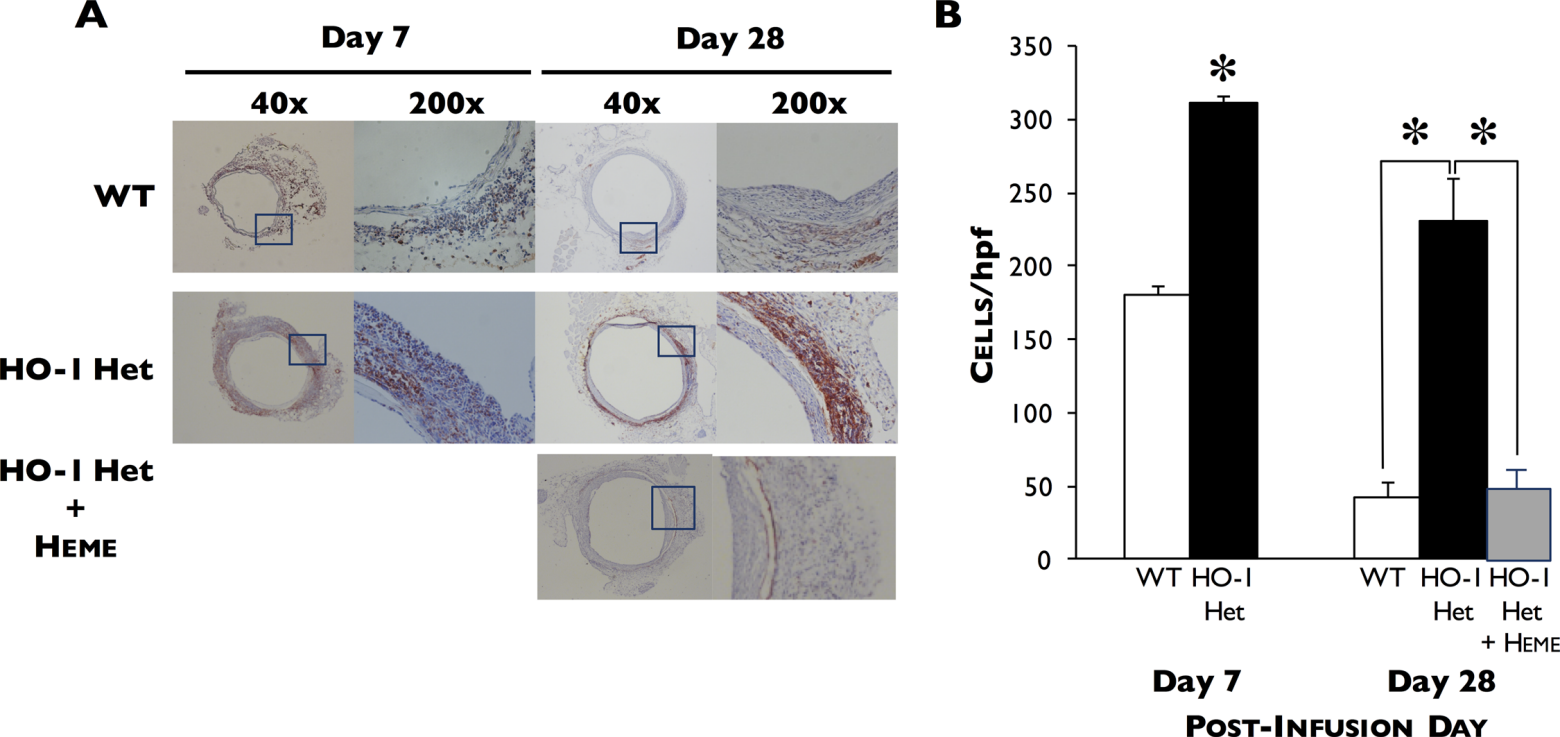
Immunohistochemical staining. (**A**) Immunohistochemical staining for Mac1 in aortae harvested from WT and HO-1 Het mice at 7 and 28 days post-PPE infusion and of heme-treated HO-1 Het mice at 28 days post-PPE infusion. (**B**) Macrophage infiltration in the aortae of WT (n = 4 and 7) and HO-1 Het (n = 4 and 7) mice at 7 and 28 days post-PPE infusion, respectively, and of heme-treated HO-1 Het mice (n = 5) at 28 days post-PPE infusion (right panel). Data shown as cells per high power field (hpf). *p<0.0001 compared to WT mice.

Immunohistochemical studies demonstrated that Mac1 staining in HO-1 Het mice was greater than that for WT mice during AAA formation, indicating an increase in the infiltration of tissue macrophages and decrease by day 28 (Fig 3A and 3B). In contrast, high levels of HO-1 staining were observed in the macrophages of WT mice 7 days post-PPE infusion (data not shown).

**Fig 4:**
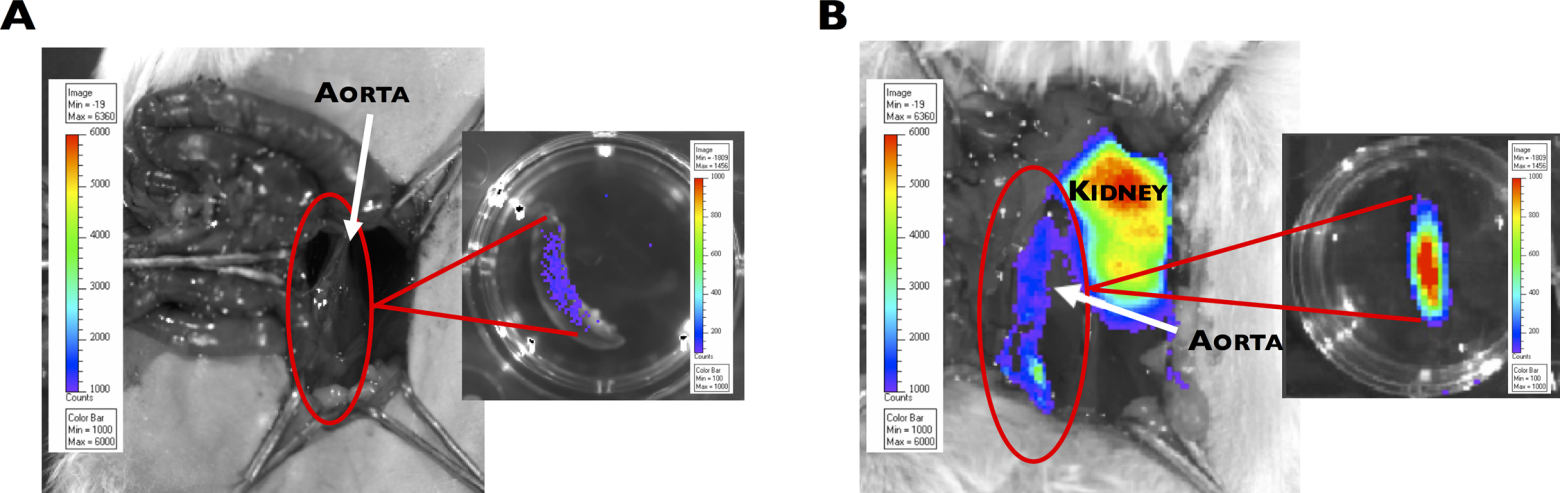
HO-1 promoter activity in the aorta following PPE infusion in HO-1-*luc* mice. *Ex vivo* images taken 14 days following (**A**) saline infusion (control) showing weak HO-1 promoter activity (low light intensity), and post-PPE infusion (**B**) showing high HO-1 promoter activity (strong light intensity) in the aorta.

Furthermore, an increase in HO-1 promoter activity in the aorta (~10-fold) was observed 14 days post-PPE infusion in HO-1-*luc* mice (Fig 4A) compared to age-matched, saline-infused control mice (Fig 4B).

### Heme injection limits experimental AAA development

Induction of HO-1 in HO-1 Het mice by IP administration of heme resulted in an increase in HO activity in livers (Fig 1A, left panel) and aortae (Fig 1B, left panel) and reduced the progression of AAA in response to PPE to a level similar to that for WT mice at 7, 14, 21, and 28 days post-PPE infusion (Fig 2). These differences began to manifest by day 7 and were significant by day 14 post-PPE infusion from that observed in untreated HO-1 Het mice. Interestingly, in heme-treated WT mice rate of AAA enlargement was slightly higher than that for heme-treated HO-1 Het and WT control mice. In addition, induction of HO-1 in HO-1 Het mice by heme administration resulted a 77% reduction (53±10 cells/hpf) in infiltrating macrophages compared to untreated HO-1 Het mice (231±79 cells/hpf, p<0.0001) at 28 days post-PPE infusion (Fig 3A and 3B).

### HO-1 deficiency results in a decreased macrophage inflammatory response

**Fig 5:**
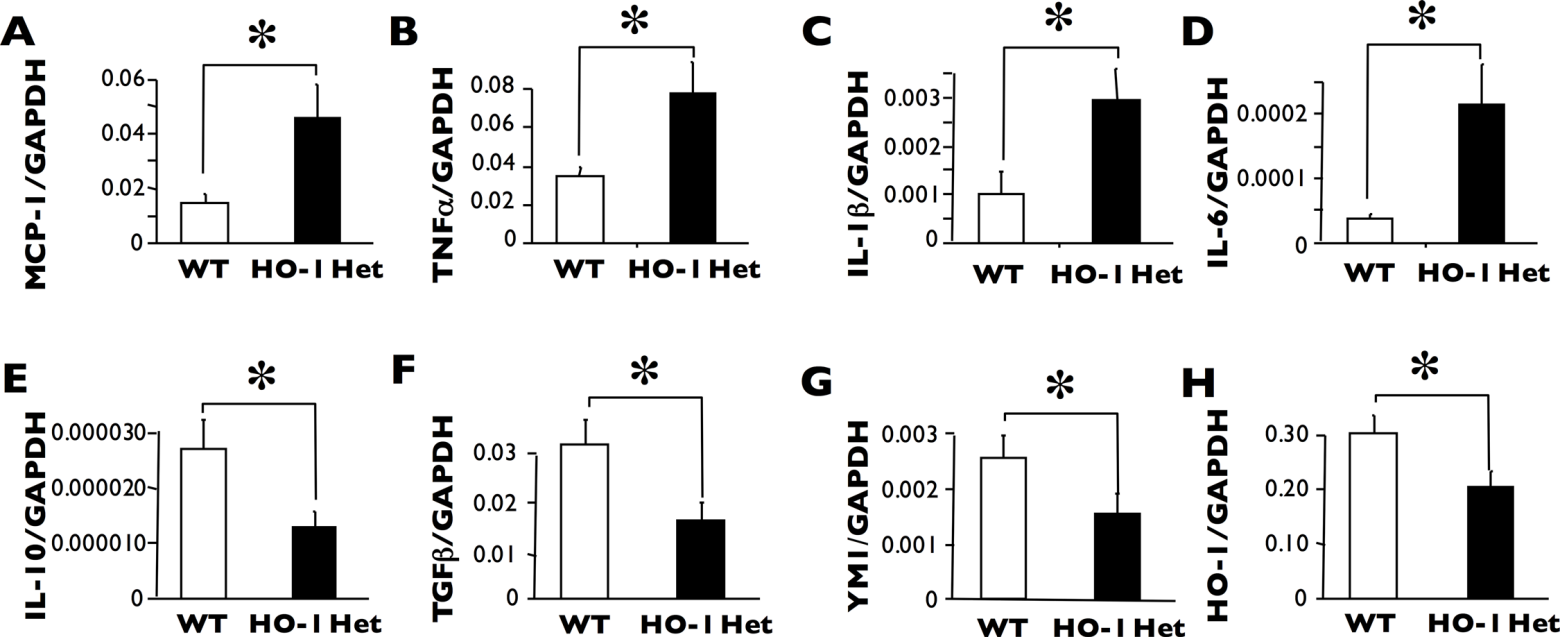
mRNA levels of the pro-inflammatory cytokines. Thioglycollate-elicited peritoneal macrophages from HO-1 Het mice exhibited significantly increased mRNA levels of MCP1 (**A**), TNF-alpha (**B**), IL-1-beta (**C**), and IL-6 (**D**) as measured using RT-PCR. In contrast, mRNA levels of anti-inflammatory cytokines IL-10 (**E**) and TGF-1 (**F**) were significantly lower. Moreover, YM-1 (**G**), one of the M2 macrophage markers, was significantly lower in HO-1 Het mice. As expected, HO-1 (**H**) levels were lower in HO-1 Het mice. *p<0.05, n = 3 to 5 for each group.

Thioglycollate-elicited peritoneal macrophages from HO-1 Het mice exhibited increased levels of mRNA expression of numerous pro-inflammatory cytokines, including MCP1, TNF-alpha, IL-1-beta, and IL-6 (Fig 5A–5D), but had decreased levels of mRNA expression of anti-inflammatory cytokines IL-10 and TGF-beta (Fig 5E and 5F) as well as the macrophage protein Ym1). As expected, HO-1 mRNA levels were decreased in HO-1 Het mice (Fig 5H).

### Statin treatment limits AAA progression

**Fig 6:**
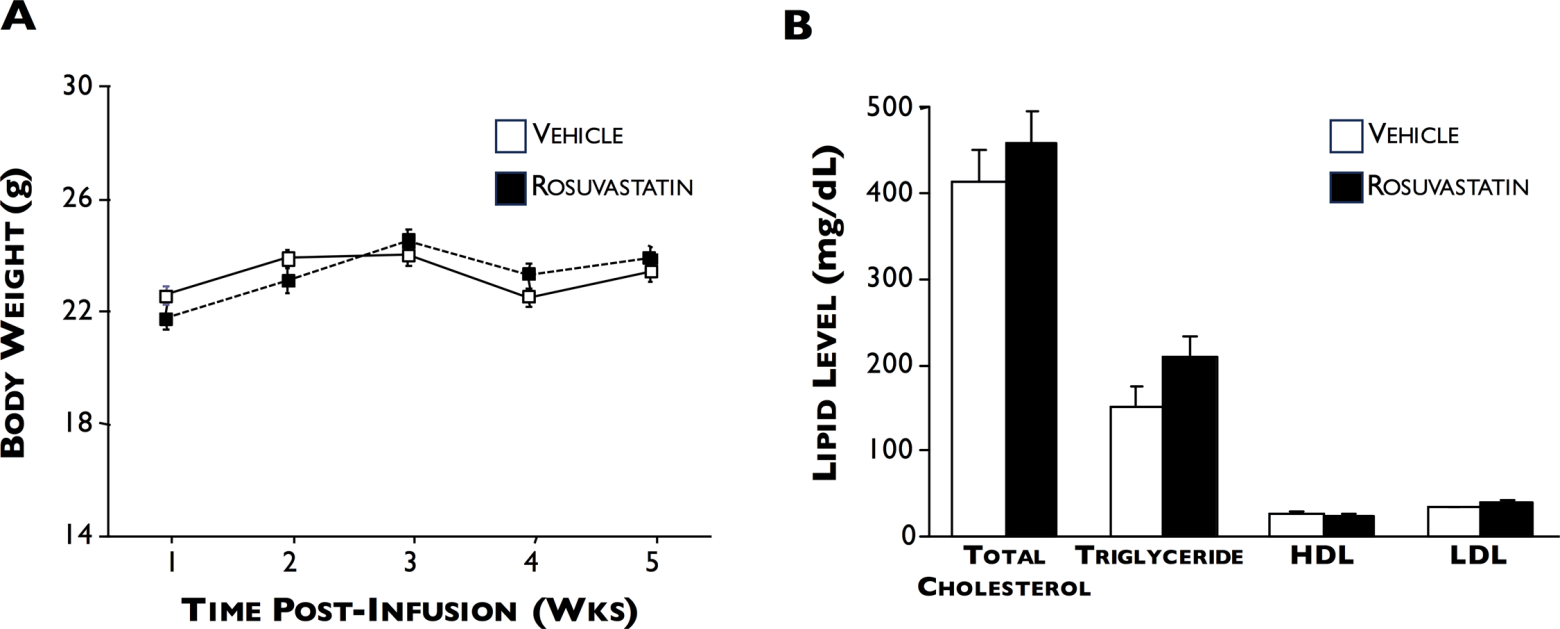
Body weight and lipid profiles. 5 wks after Ang II infusion, body weight (**A**) and lipid (**B**) profiles were measured in vehicle- (white squares, n = 10) and rosuvastatin- (black squares, n = 9) treated ApoE^-/-^ mice. No significant differences were found between the 2 groups.

**Fig 7:**
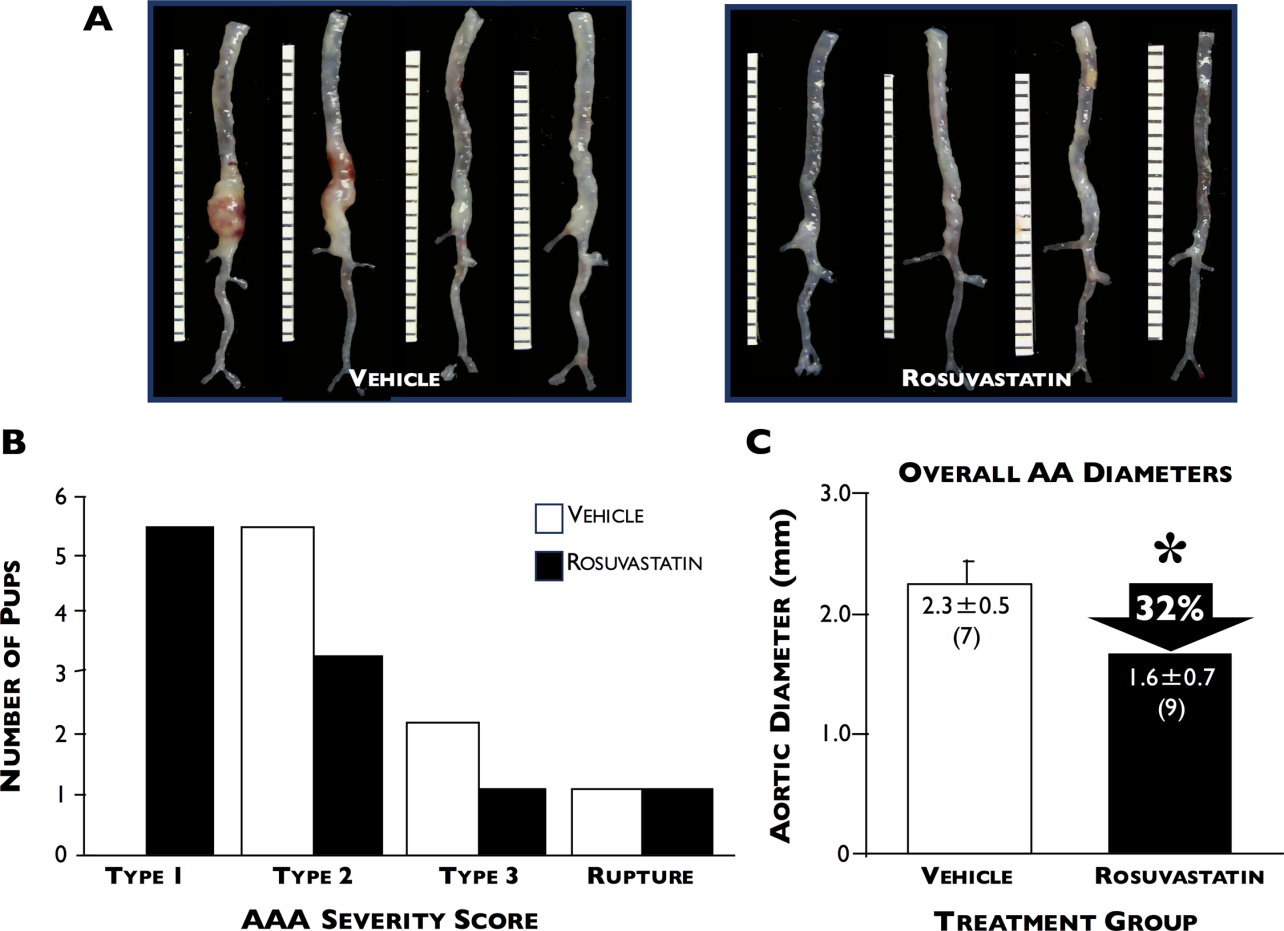
Effect of rosuvastatin on AAA severity. (**A**) Representative *ex vivo* pictures of aortas harvested from ApoE^-/-^ mice treated with saline-vehicle (left panel) or rosuvastatin (right panel) following Ang II infusion. (**B**) Number of pups categorized by AAA severity scores post-Ang II infusion in vehicle- (white squares) or rosuvastatin- (black squares) treated mice. (**C**) Overall AAA severity in both groups combined. *p = 0.04, n = 7 and 9 for vehicle- and rosuvastatin-treated mice, respectively.

**Fig 8:**
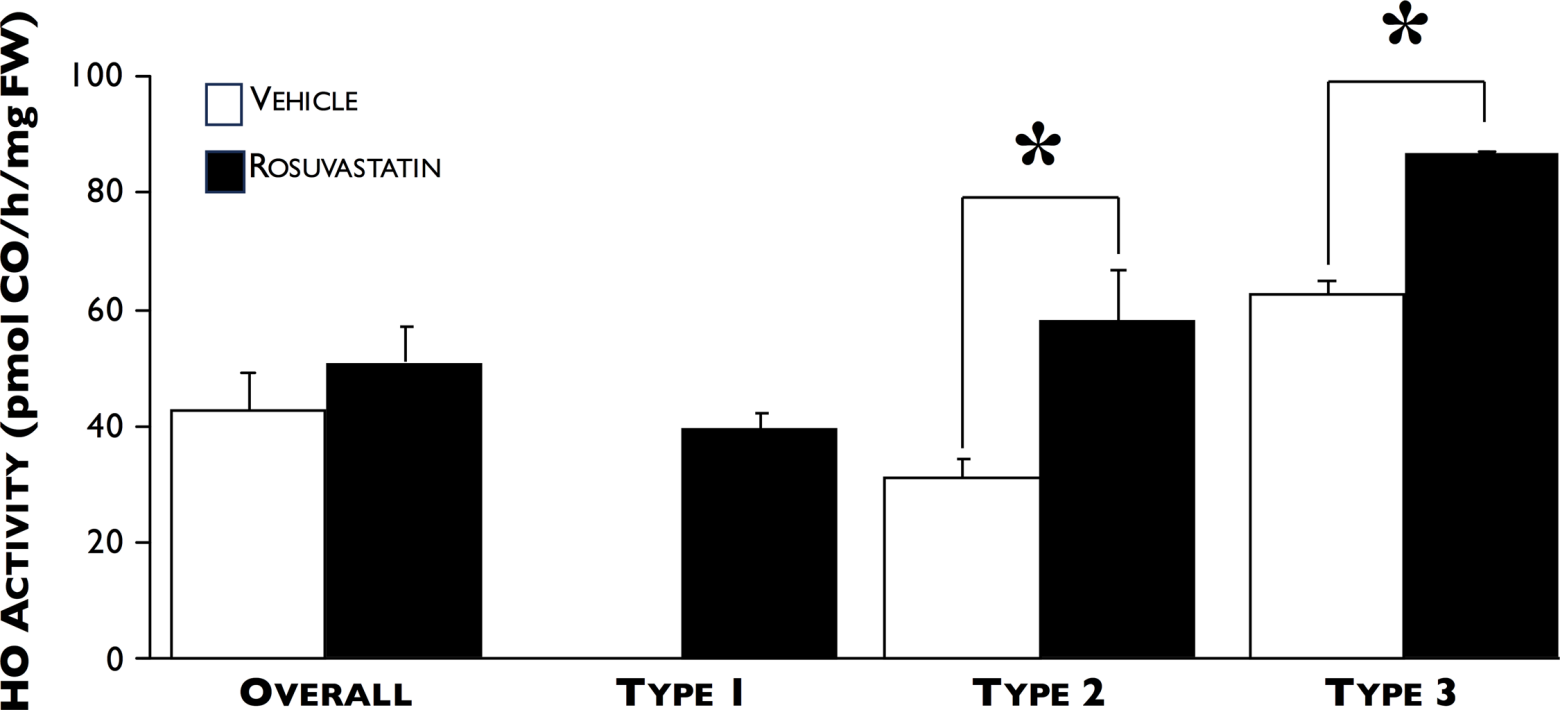
Effect of rosuvastatin on HO activity and AAA severity. No significant difference in HO activity between aortas from vehicle- (white squares) and rosuvastatin- (black squares) treated ApoE^-/-^ mice were found 14 days post-Ang II infusion, but when grouped by severity, HO activity in aortas with type-2 expansions was significantly higher in rosuvastatin-treated mice. *p<0.05, n = 3 for each group.

Statin and vehicle-treated ApoE^*-/-*^ mice did not differ in their body weights or lipid profiles (including total cholesterol, triglycerides, HDL, and LDL) at the end of the time-course (Fig 6). Fig 7 shows that rosuvastatin treatment significantly reduced the size of AAA development by 32% compared to vehicle only-treated (2.3±0.2 versus 1.6±0.2 mm, respectively, p<0.04). When total HO enzyme activity was measured in the aortae of vehicle- and rosuvastatin-treated mice, no significant difference was found between the two groups (42.6±19.1 (n = 6) versus 51.2±19.2 pmol CO/h/mg FW (n = 9), respectively, Fig 8). However, when AAA was categorized by severity, we found that HO activity following type 2 and 3 AAA expansions were significantly higher in the rosuvastatin group (31.0±9.7 versus 58.1±19.1 and 65.9 versus 86.6 pmol CO/h/mg FW, respectively, p<0.05).

## Discussion

The etiology of AAA formation is complex and multifactorial [29]. Prior studies have shown that AAA development is associated with a profound inflammatory response and resultant degradation of the extracellular matrix [29–31]. In the PPE murine AAA model, it is this inflammatory process, and not the infusion of PPE itself, that leads to aneurysmal dilatation [32]. Not surprisingly, administration of drugs that target specific points in the inflammatory cascade have been shown to reduce AAA progression in experimental models–but thus far this has not been shown in patients with AAA disease [33–35].

Studies of HO-1 knockout mice and human HO-1 promoter polymorphisms have demonstrated that HO-1 is central to the antioxidant defense of vascular tissues [36–38]. As a result, several studies have investigated the role of HO-1 in relation to AAA. In humans, Schillinger et al [3] reported that the length of a (GT)n repeat in the HO-1 gene promoter modulates the level of HO-1 gene transcription, with short (GT)n repeats associated with increased HO-1 expression in response to inflammatory stimuli. They found that fewer AAA patients tended to have these beneficially shorter repeats when compared to age- and sex-matched patients with coronary or peripheral artery disease. Although a decreased ability to upregulate HO-1 may be a risk factor, it is likely that in most cases, other conditions (genetic or environmental) that may predispose an individual to a pro-inflammatory disposition, would in combination increase risk. In fact, the presence of a stressor may be required to reveal this phenotype.

In a preclinical animal model of AAA disease, we previously showed that increases in local blood flow results in smaller AAA diameters, and microarray analyses and confirmation by RT-PCR indicated that HO-1 is significantly upregulated during this process. In addition, flow loading has been shown to result in reduced ROS production [12]. These results are of interest because of the reported role of HO-1 in oxidative stress [39]. Heme degradation by HO leads to the generation of biliverdin, which is converted by biliverdin reductase to form bilirubin [40]. Biliverdin and bilirubin have been shown to be efficient scavengers of ROS and are important to cellular defense mechanisms against oxidative tissue injury [41,42]. Likewise, CO, another HO byproduct, is a well-known anti-apoptotic, anti-inflammatory, and anti-proliferative molecule that has been investigated for protecting against injury in the cardiovascular and respiratory systems [6,37]. For these reasons and because HO-1 is highly conserved and widely expressed in human and animal cells, we hypothesized that it could serve as a therapeutic target.

Using the PPE model, we found that HO-1 was induced during AAA formation in WT mice, but significantly less so in HO-1-deficient (Het) mice. In addition, HO-1 Het mice showed an augmented development of AAA and an increased local infiltration of macrophages. Peritoneal macrophages isolated from these mice showed the expected diminished HO-1 expression as well as an increased expression of inflammatory cytokines and a decreased expression of protective cytokines. Moreover, induction of HO-1 expression in HO-1 Het mice via heme treatment increased HO activity to near WT baseline levels and attenuated AAA development, with aneurysm growth profiles approaching that seen for WT mice. Furthermore, heme treatment also reduced macrophage infiltration in AAA-induced HO-1 Het mice, highlighting that the induction of HO-1 leads to anti-inflammatory responses and reduces a pro-inflammatory response. Interesting, we observed that heme treatment to WT mice did not attenuate AAA enlargement, and in fact, AAA size approached that of HO-1 Het levels. This may be due to the fact that an over-induction of HO-1 may be detrimental as well as the fact that the high dose of heme used may have been toxic. In contrast, heme treatment to HO-1 Het mice may was able to induce HO-1 expression to basal levels observed in WT mice and provided protection.

Several studies have demonstrated a beneficial effect of statins in animal AAA models. Simvastatin was shown to decrease both AAA incidence and aortic diameters in both WT and ApoE^-/-^ mice following PPE perfusion [43]. This statin was also shown to decrease AAA diameters, MMP-9 and NF-kappa B protein levels, and microarray gene clusters related to inflammation, extracellular matrix remodeling, and oxidative stress function in PPE-perfused rats [44]. In humans, it has been shown that over a median period of 3.1 yrs in patients diagnosed with AAAs, statin use resulted in a 1.16 mm/yr lower AAA diameter growth rate compared to non-statin users [45]. Protein analyses of AAA aortic walls in patients randomized to simvastatin therapy before undergoing open repair had lower levels of MMP-3 and MMP-9 [46]. Cellular analyses of their AAA walls further confirmed that statin use significantly reduced MMP9 levels [47,48]. Notably, these clinical findings are considered controversial, since these positive outcomes have not been replicated in larger and more rigorously performed studies. Still, these overall findings in both human and experimental models of AAA disease do suggest that statins may have potential to beneficially affect the pathogenesis of AAA.

Statins are well known to have pleotropic, tissue-protective effects in a manner independent of its cholesterol-lowering properties. We have previously shown that HO-1 is a functionally relevant target of several structurally different statins in endothelial cells suggesting a class effect of these HMG-CoA reductase inhibitors. For all statins tested, an increase in HO-1 expression was observed and verified at both the transcriptional and translational levels [49,50]. For simvastatin, induction of HO-1 protein in vascular smooth muscle cells was reported by Lee et al [51]. HO-1 induction and associated biological effects, such as antioxidant tissue protection occurred at clinically effective concentrations of statins. As a consequence of this genomic action, cells were protected from oxidative stress [49,50]. Unlike other pleiotropic effects of statins, HO-1 induction does not seem to depend on the inhibition of HMG-CoA reductase and decreased formation of isoprenoids that are important for small G-protein activation [19]. In these studies, the addition of the HMG-CoA product mevalonate or the isoprenoid farnesylpyrophosphate did not attenuate the response of HO-1 to statins [49,50], precluding the involvement of isoprenoid-dependent pathways such as blockade of Rho and its downstream target Rho kinase [52,53]. Based on this evidence, we believe that statin-dependent HO-1 induction occurs in vascular and non-vascular tissues in vivo.

Our study indicates that some of these protective properties of statins likely derive from their ability to effectively induce HO-1 expression. Rosuvastatin administration reduced AAA progression in the Ang II model, and increased HO enzyme activity, independent of changes in lipid profile. These results are consistent with our previous findings that statin administration induces HO-1 in heart and lung tissues [23]. Taken together, these data suggest that the suppression of AAA progression with statin administration is mediated, at least in part, by some of the cytoprotective properties of HO-1. Future studies elucidating if the mechanism is mediated by the effects of HO-1 alone or via other anti-inflammatory or antioxidative processes is warranted and beyond the scope of these studies.

In summary, we provide evidence of a crucial role of HO-1 in limiting aneurysm growth by showing that HO-1 induction by heme slows AAA progression, confirming prior reports of suppression of AAA progression with statin administration, and demonstrating that the statin therapeutic mechanism likely involves the induction of HO-1 and likely independent of lipid lowering. Therefore, development of specific targeted therapies that induce HO-1 expression may be a new therapeutic strategy for the management of AAA disease as well as other inflammatory vascular diseases, such as atherosclerotic disease in other vessels.
